# Impact of the Direct Ageing Procedure on the Age Hardening Response of Al-Mg-Si 6101 Alloy

**DOI:** 10.3390/ma11071239

**Published:** 2018-07-19

**Authors:** Piotr Osuch, Monika Walkowicz, Tadeusz Knych, Stanislaw Dymek

**Affiliations:** 1 Department of Metal Working and Physical Metallurgy of Non-Ferrous Metals, Faculty of Non-Ferrous Metals, AGH University of Science and Technology, al. A. Mickiewicza 30, 30-059 Krakow, Poland; monika.walkowicz@agh.edu.pl (M.W.); tknych@agh.edu.pl (T.K.); 2 Department of Surface Engineering & Materials Characterisation, Faculty of Metals Engineering and Industrial Computer Science, AGH University of Science and Technology, al. A. Mickiewicza 30, 30-059 Krakow, Poland; dymek@agh.edu.pl

**Keywords:** aluminum alloys, precipitation kinetics, electrical resistivity, hardness measurement, electron microscopy, age hardening, phase transformation, precipitate-free zones

## Abstract

Al-Mg-Si alloys are used not only as construction material, but also as a material for electrical conductors. For this application, it is crucial for the alloy to achieve a balance between strength and electrical properties. This is achieved in practice by a combination of strain and precipitation hardening. The current paper focuses on a heat treatment procedure in which the EN AW 6101 alloy is cooled by a flowing air stream from the solutionizing temperature down to the artificial ageing temperature. The proposed procedure, unlike the common heat treatment leading to the T6 temper, allowed for the precipitation of the coarser β” phase with the presence of relatively wide precipitate-free zones. The age hardening response was investigated by Brinell hardness measurements, eddy current testing and microstructural observations using transmission electron microscopy (TEM). The applied heat treatment resulted in slightly lower strength (compared to the T6 temper), but improved electrical performance of the alloy.

## 1. Introduction

Due to a high strength-to-weight ratio, good electrical conductivity and corrosion resistance, Al-Mg-Si alloys (EN AW 6xxx series alloys) are widely used in electrical applications, mainly for overhead power cables. In this case, manufacturing technologies that usually take advantage of a combined effect of strain and precipitation hardening in order to increase strength should also enhance the electrical performance of the final product. This can be achieved through an appropriately-designed heat treatment procedure.

An important role of the 6xxx alloys’ heat treatment pertains to the precipitation kinetics of particular metastable phases from a supersaturated solid solution. In terms of nucleation and growth mechanisms, precipitates in the 6xxx series alloys can be divided into two categories: homogeneous (atomic clusters, GP zones, β”) [1,2,3,4,5,6,7,8,9,10,11,12,13,14,15,16,17,18] and heterogeneous (β’, U1, U2, B’, β) [19,20,21,22,23,24]. Homogeneous precipitates nucleate from a supersaturated solid solution uniformly in the whole volume of the supersaturated aluminum matrix α. They nucleate at relatively low temperatures; their crystal structures are mostly coherent with the matrix; and they exhibit relatively low interfacial energy [1,2,3,4,5,6,7,8,9,10,11,12,13,14,15,16,17,18]. For instance, atomic clusters nucleate at room temperature, while GP zones need elevated temperatures just below 100 °C to nucleate [6,7,8,9,10,11,12,13,14,15,16]. The main hardening precipitates, β”, nucleate at temperatures above 100 °C and remain metastable up to temperatures well below 200 °C [17,18,19,20,21,22,23]. 

However, the precipitation of solute atoms in such alloys often occurs heterogeneously on various, non-equilibrium defects such as vacancies, dislocations, grain boundaries or second-phase particles [1]. The precipitates formed by heterogeneous nucleation are semi-coherent or non-coherent with the aluminum matrix; their interfacial energies are high, and therefore, they preferentially nucleate at higher temperatures, usually above 200 °C [18,19,20,21,22,23,24]. The nucleation rate of these particular precipitates is different, and it increases with temperature. 

**Figure 1:**
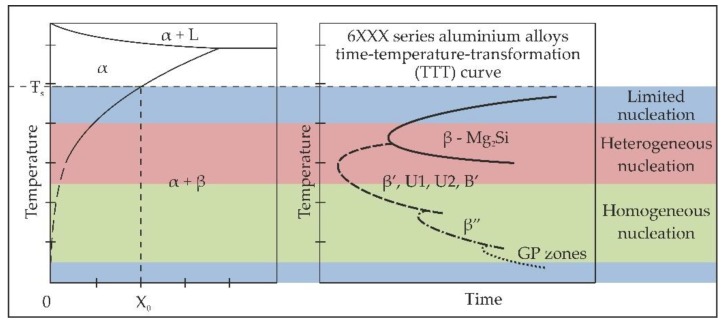
Scheme of the precipitation kinetics in 6xxx series alloys.

At room temperature, the nucleation rate is relatively low because of low atom mobility. The achievement of a stable level of mechanical and electrical properties upon natural ageing of a 6xxx aluminum alloy usually requires several days of exposure [6,7,8,9,10,11,12,13,14,15,16]. The nucleation of GP zones at temperatures of about 100 °C takes several dozens of hours [6], and nucleation of the metastable β” precipitates usually requires artificial ageing for over a dozen hours [17,18,19,20,21,22]. However, the nucleation of one of the heterogeneous, metastable phases occurs in less than one hour at temperatures exceeding 250 °C [19,20,21,22,23,24]. On the other hand, at extremely high temperatures, just below the solvus line, the driving force for precipitation is limited because of the small difference in solute atom concentration, and the interfacial energy for the nucleation of large, non-coherent, equilibrium precipitates increases; thus, the nucleation rate decreases [1]. The precipitation kinetics in the 6xxx series alloys can be demonstrated on time-temperature-transformation (TTT) curves in relation to the Al-Mg_2_Si pseudo-binary phase diagram and the variation of enthalpy and precipitation rate with undercooling, as is shown in Figure 1.

In the presented TTT diagram (Figure 1 right side), specific points on the curves represent a combination of temperature and time of exposure after which a given type of precipitate can be referred to as metastable (end of transformation at the given temperature). This TTT diagram is only illustrative because the nucleation rate of particular metastable phases depends on numerous factors, such as concentration of solute atoms in solid solution in the case of the homogeneous nucleation, or the concentration of heterogeneous nucleation sites in the case of the heterogeneous nucleation. It should also be pointed out that nucleation of precipitates, especially homogeneous ones, is controlled by an excess of vacancies, which are responsible for the increasing diffusion rate of solute atoms at ageing temperatures [25]. Vacancies are also responsible for the formation of precipitate-free zones (PFZ) in the vicinity of grain boundaries and other heterogeneous sites due to their fast diffusion to such defects. The PFZs can also be formed as a result of solute depletion in the vicinity of grain boundaries and other heterogeneous sites.

Because of these phenomena, industrial processes of solution heat treatment and quenching of 6xxx series alloys are usually designed in such a manner so as to avoid heterogeneous nucleation, as well as the formation of PFZs, thus maximizing the tensile strength of the final product. However, in electrical applications, in which particular emphasis is put on the resistivity of the final product, the procedures of heat treatment are designed to overage the alloy intentionally. The overaging makes the concentration of solute atoms in the matrix smaller. The solute atoms diffuse not only into homogeneous precipitates, but to some extent, into heterogeneous ones, and thus, the electrical performance can be improved with only a slight decline in the mechanical properties. In the current paper, a heat treatment procedure is proposed, in which the 6101 series alloy was cooled down from the solutionizing temperature directly to the artificial ageing temperature, followed by holding the alloy at the temperature for a certain period of time. The purpose of this work is to investigate the influence of such a heat treatment procedure on the microstructure, mechanical and electrical properties of the 6101 alloy in comparison with the conventional heat treatment leading to the T6 temper (solution heat treated then artificially aged).

## 2. Materials and Methods 

The chemical composition of the EN AW 6101 wrought aluminum alloy used in this study is listed in Table 1. 

**Figure 2:**
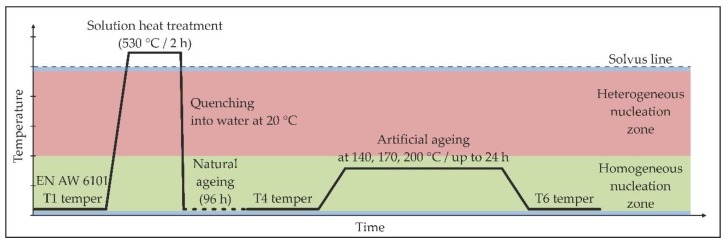
Temperature-time scheme of the conventional heat treatment procedure to the T6 temper.

The material was manufactured in industrial continuous casting and rolling processes into the form of a 9.5 mm-diameter rod in a T1 temper (cooled from the elevated temperature shaping process and naturally aged) in accordance with the EN 1715-2 standard. Samples excised from the as-received material were 10 mm in length. Two different heat treatment procedures were performed to investigate their effect on the microstructure, mechanical and electrical properties of the alloy. The first group of samples was solution heat treated for 2 h at 530 °C, quenched in water at 20 °C, naturally aged at the room temperature for 96 hours and then isothermally aged at the temperatures of 140, 170 and 200 °C with different ageing periods in a forced-air oven. This reflects the typical heat treatment method for this type of alloy, firstly to T4 temper and then to T6 temper. The schematic representation of the first type of the heat treatment procedure is shown in Figure 2.

**Figure 3:**
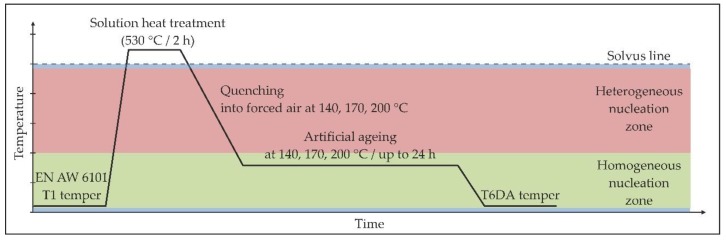
Temperature-time scheme of the direct ageing procedure (T6DA).

The second group of samples were also solution heat treated at 530 °C for 2 h and then immediately transferred to a forced-air furnace with temperatures set to 140, 170 and 200 °C. The samples were held at these temperatures for different ageing periods. This procedure is referred to as the direct ageing (DA) procedure. The schematic representation of such a heat treatment is shown in Figure 3.

In order to monitor the actual temperature changes in time during these heat treatments, thermocouples were placed inside drilled holes in some of the samples, and measurements were recorded at a frequency of 1 Hz. Brinell hardness measurements (HBW 2.5/31.25) were performed to investigate an age hardening response. Two different samples were measured with three indentations symmetrically placed around a central axis of slices cut off from the sample (rod). Thus, the data points on the hardness curves were averages from 6 indents. A maximum standard deviation of 2.1 HBW 2.5/31.25 was achieved. The electrical resistivity was measured at room temperature using an eddy-current electric conductivity meter (SIGMATEST 2.069, Foerster Instruments, Pittsburgh, PA, USA). The device was equipped with a measuring probe with a diameter of 8 mm, which covered almost the whole area of the samples’ cross-sections. The frequency of electrical current induced during the measurements was set to 960 kHz. This, according to the device specification, provided a measurement depth of 1.2 mm under the sample surface. 

Microstructural observations of longitudinal sections of the heat-treated samples were carried out using scanning and transmission electron microscopes (SEM-HITACHI S-3500N, Hitachi, Chiyoda, Tokyo, Japan and TEM-JEOL JEM-2010, JEOL, Akishima, Tokyo, Japan) together with chemical analysis utilizing energy-dispersive spectroscopy (EDS). TEM foils in the form of 3-mm disks were prepared by mechanically grinding to about 0.1 mm followed by jet thinning with an electropolishing unit.

## 3. Results

**Figure 4:**
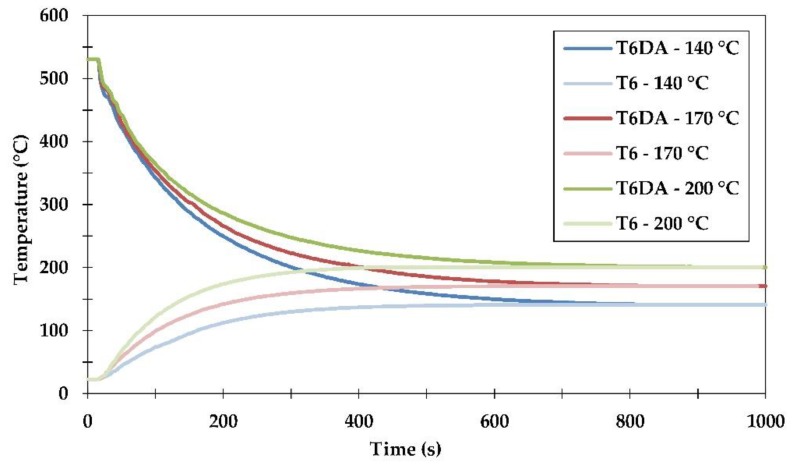
Temperature evolution measured during alloy quenching from a solutionizing temperature of 530 °C directly to artificial ageing temperatures of 140 °C, 170 °C and 200 °C (T6DA procedure), as well as during heating to artificial ageing temperatures from room temperature (T6 temper procedure).

The conventional procedure of sample quenching from the solutionizing temperature of 530 °C to room temperature lasted less than one second. This is below the measurement resolution capability used in the current study. However, achieving the desired level of artificial ageing temperature during both investigated heat treatment procedures certainly required more time and could be monitored. The results showing the temperature changes with time are presented in Figure 4. 

It was found that samples cooled down directly from the solutionizing temperature to the ageing temperatures of 140, 170 and 200 °C reached the final temperatures after ≈800 seconds. It should be pointed out that during this DA procedure, the samples were exposed to temperatures in the range of about 200–400 °C for a prolonged period of time. This stimulated a depletion from vacancies and created the most preferred conditions for heterogeneous nucleation of second phase precipitates. As might have been expected, the exposure time for this temperature range decreased with the quenching/artificial ageing temperatures and equaled ≈240 s, ≈340 s and over 800 s for quenching temperatures of 140 °C, 170 °C and 200 °C, respectively.

The measurements of temperature change with time during the conventional procedure of artificial ageing to the T6 temper were also performed. It was found that reaching the artificial ageing temperatures of 140 °C, 170 °C and 200 °C from room temperature took between 400 and 500 s. Taking into consideration that the diffusion of atoms was relatively slow in the temperature range between room temperature and about 200 °C, the impact of the alloy exposure to changing temperatures was relatively low to none as compared to the prolonged exposure during undercooling in the DA procedure.

**Figure 5:**
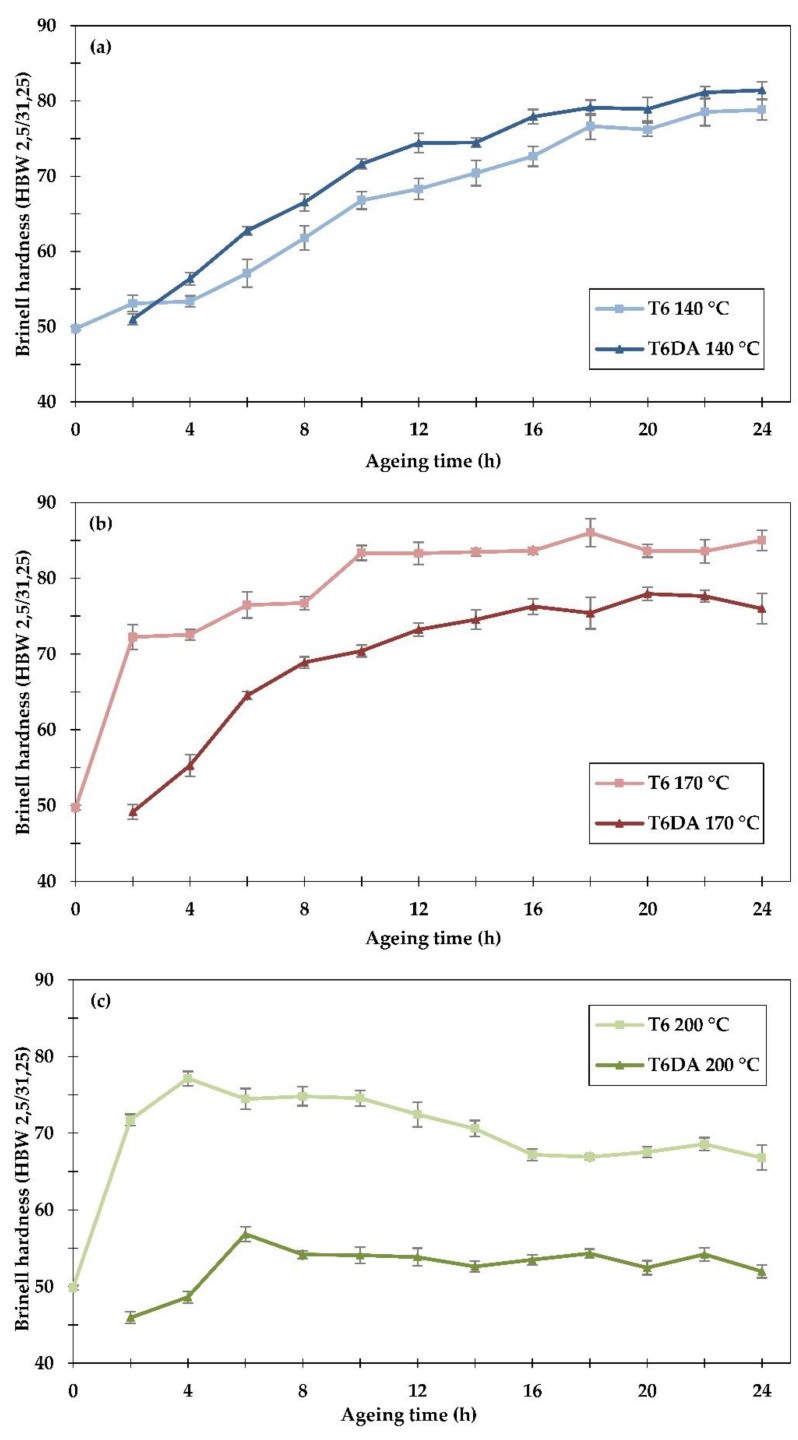
Brinell hardness (HBW 2.5/31.25) of the samples subjected to artificial ageing at (**a**) 140 °C, (**b**) 170 °C and (**c**) 200 °C, using two different heat treatment procedures: to the conventional T6 temper and directly to the ageing temperature.

The age hardening responses of the examined samples after both investigated heat treatment procedures were measured by the Brinell hardness method, and the results are shown in Figure 5. 

The results have been collected on separate graphs, accordingly to the ageing temperature, in order to compare the applied heat treatment procedures. When ageing at the temperature of 140 °C, the Brinell hardness gradually increases with the ageing time, reaching ≈78.5 HBW after 24 h of ageing in the conventional heat treatment procedure and ≈81.5 HBW after 24 h of ageing directly from the solutionizing temperature. The hardness of the alloy aged directly from the solutionizing temperature is higher after almost every ageing time interval. The opposite relation can be observed at the ageing temperature of 170 °C, where after the conventional ageing, the alloy reached its peak hardness after 18 h at ≈86 HBW, while after the DA procedure, the hardness was slightly lower and reached ≈78 HBW after 20 h of ageing. A similar relation can be observed at 200 °C, where in conventional ageing to the T6 temper, the alloy reached the peak hardness at ≈77 HBW after just four hours of ageing, while in the DA procedure, the hardness was lower and reached ≈57 HBW after six hours of ageing. It should be noted that the DA procedure is able to provide a similar level of hardness compared to the conventional method. At 140 °C, the DA procedure resulted in higher values of hardness. This can be rationalized by the fact that the temperature of 140 °C can be considered as relatively low, since after a maximum ageing time of 24 h, the alloy has not reached its peak hardness. Thus, exposing the alloy to elevated temperatures during the undercooling procedure increased the nucleation rate during the initial stage of artificial ageing, resulting in enhanced strength of the alloy compared to the T6 temper procedure. On the other hand, at temperatures of 170 °C and 200 °C, an adverse effect of the prolonged exposure to elevated temperatures (200–400 °C) in the DA procedure was observed. 

**Figure 6:**
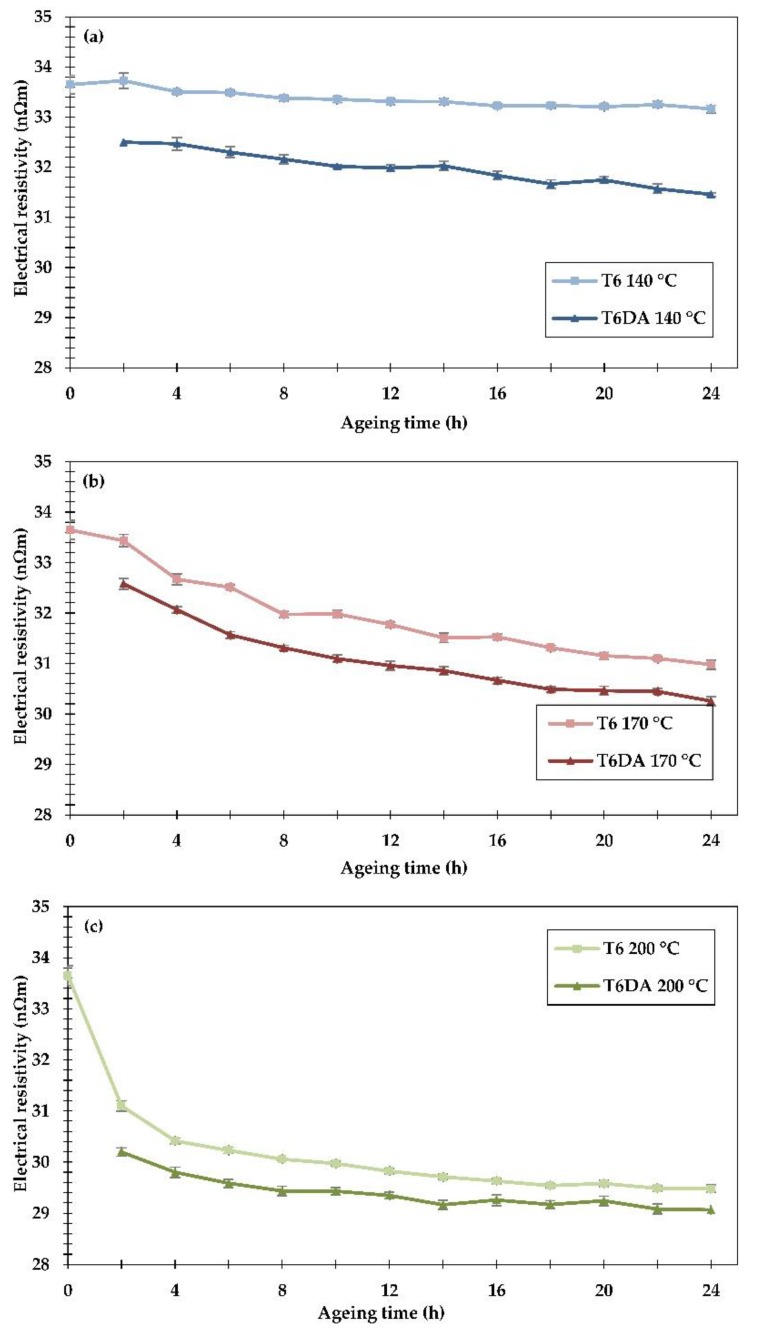
Electrical resistivity of the EN AW 6101 wire rod subjected to artificial ageing at (**a**) 140 °C, (**b**) 170 °C and (**c**) 200 °C, using two different heat treatment procedures: to the conventional T6 temper and directly to the ageing temperature.

The electrical resistivity measured at room temperature of the examined alloy subjected to both heat treatments is shown in Figure 6. For the sake of easier comparison, the results are grouped in separate graphs according to the ageing temperatures. 

In the conventional heat treatment to the T6 temper, the resistivity decreased only slightly (about 1.4%) after 24 h of ageing at 140 °C, reaching the value of 33.2 nΩm. On the other hand, during the DA procedure, the resistivity decreased significantly: 3.4% at the first measurement point, just after two hours of ageing; and it continued to decrease gradually, achieving resistivity values 6.5% lower than in non-aged samples (31.5 nΩm) and about 5% lower than after the conventional ageing to the T6 temper lasting 24 h. These results show that during the undercooling stage, evident phase transformations occurred in which solute atoms diffused out of the matrix, leading to a substantial drop in resistivity. It should also be pointed out that phase transformations taking place during undercooling affected the phase transformations occurring during subsequent artificial ageing, leading to more a substantial drop in resistivity than in the conventional method. At the ageing temperature of 170 °C the DA procedure provided lower resistivity after every time interval compared to the conventional procedure. In the early stage of artificial ageing, a significant decrease (−3.2%) in resistivity can be observed in the DA procedure just after two hours of ageing. After 24 h of ageing in the DA procedure, the drop in resistivity exceeded 10%, while in the conventional heat treatment, the resistivity decreased by ≈8%, achieving 30.3 nΩm and 31 nΩm, respectively. At the ageing temperature of 200 °C, the resistivity decrease was noticeable after just 2 h of artificial ageing during both investigated heat treatments. During the longer ageing time intervals the resistivity somewhat decreased, as the driving force for phase transformations became weaker at this relatively high temperature. Using the DA procedure with 24 hours of ageing time, the resistivity reached ≈29 nΩm, while in the conventional method, it was ≈29.5 nΩm.

**Figure 7:**
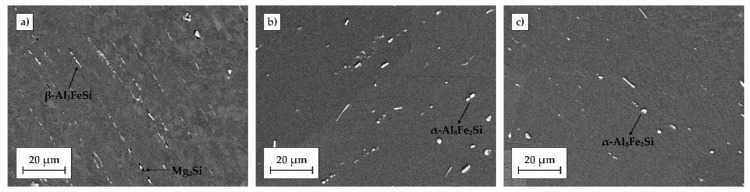
SEM micrographs of longitudinal sections of the wire rod after different heat treatment stages: (**a**) as-fabricated; (**b**) solution heat treated at 530 °C/2 h, water quenched, naturally aged, artificially aged at 170 °C/24 h; (**c**) solution heat treated at 530 °C/2 h, cooled down by flowing air forced to 170 °C, artificially aged at 170 °C/24 h.

In order to investigate the phase transformations that were responsible for the macroscopic effects described above, longitudinal sections of the alloy rods in different tempers were examined using SEM (see Figure 7). In the microstructure of the rod in the as-fabricated condition, second phase particles, likely Mg_2_Si and α-Al_5_FeSi, were identified upon their chemistry determined by the EDS method. These particles are reported to be typical ones that crystalize from melt during solidification in the industrial processes of the 6xxx alloys. The typical particles are shown in Figure 7a. It should also be pointed out that all large particles are situated along the rolling direction as a result of plastic deformation in a multi-stand rolling mill. After solutionizing treatment, followed by quenching in water (Figure 7b) or quenching directly to the ageing temperature, followed by artificial ageing for 24 h at 170 °C (Figure 7c), it was found that Mg_2_Si particles were completely dissolved in the matrix.

**Figure 8:**
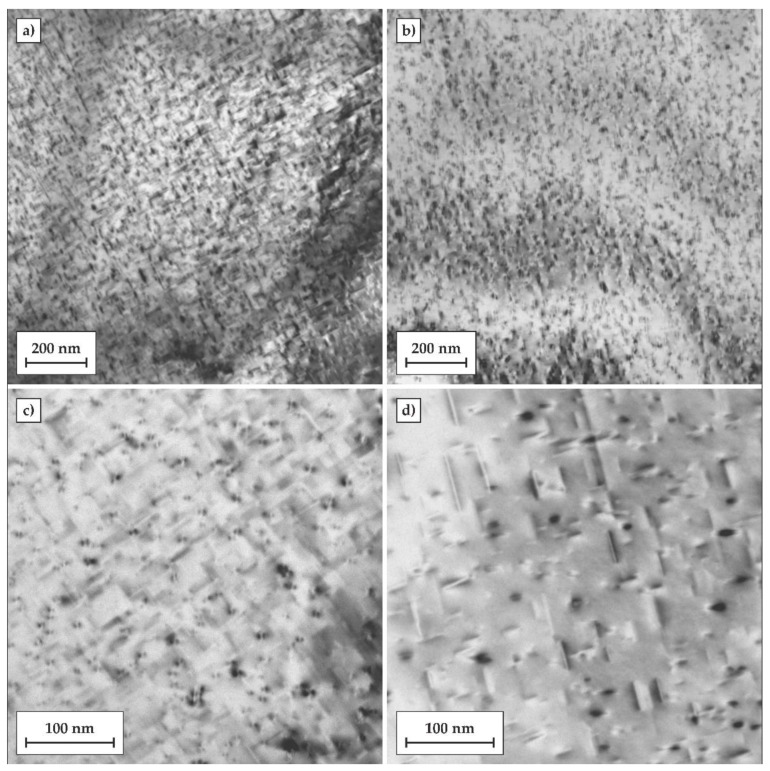
TEM micrographs showing the matrix precipitation of the alloy after two investigated heat treatment procedures: (**a**,**c**) conventional heat treatment to the T6 temper including artificial ageing at 170°C for 24 h; (**b**,**d**) quenched directly to the temperature of 170 °C followed by 24 h of artificial ageing (T6DA).

The microstructural evolution in the Al matrix after both heat treatments was investigated by TEM. Samples after 24 h of artificial ageing at the temperature of 170 °C were selected for TEM observations. The results are shown in Figure 8. Based on previous studies and the current TEM observations, the needle-shaped precipitates β” can be identified in the aluminum matrix. In the T6 temper, the number density of β″ precipitates was significantly higher, and their sizes were also much finer compared to those of the directly-aged T6 (DA) temper. This may be associated with the fact that the atomic clusters formed during natural ageing may act as preferred nucleation sites for the β″ precipitates and that their further growth and coarsening was controlled by the Ostwald ripening phenomenon during artificial ageing. On the other hand, the cooling step, from the solutionizing temperature to the artificial ageing temperature, provided prolonged exposure of the alloy at elevated temperatures, introducing a larger driving force for direct formation of much coarser and more widely-dispersed β″ precipitates. 

**Figure 9:**
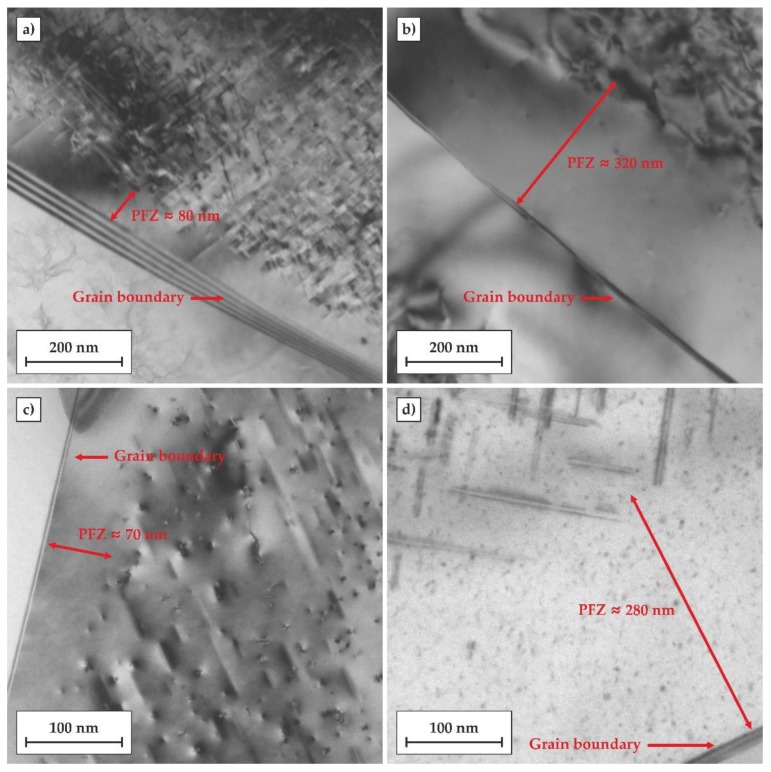
TEM micrographs showing grain boundary regions of the alloy after two investigated heat treatments: (**a**,**c**) conventional heat treatment to the T6 temper including artificial ageing at 170 °C for 24 h; (**b**,**d**) quenched directly to the temperature of 170 °C followed by 24 h artificial ageing (T6DA). PFZ, precipitate-free zones.

Figure 9 shows TEM micrographs of grain boundary regions in the alloy subjected to both investigated heat treatments comprised of artificial ageing for 24 h at 170 °C. The width of the PFZs in the samples subjected to the regular T6 treatment was about 50–100 nm, while the PFZs in the T6DA temper were significantly wider and reached a width of about 250–300 nm. This shows that the prolonged exposure of the alloy to elevated temperatures caused the diffusion of vacancies to grain boundaries, impeding the nucleation of precipitates near grain boundaries during the cooling step in the DA treatment (ca. 200–400 °C). At the same time, it should be noted that there was no evidence of grain boundary precipitates (GDP) formed in this process. GDPs are generally undesirable since they are regarded as a principal cause of intergranular corrosion [26,27]. 

**Figure 10:**
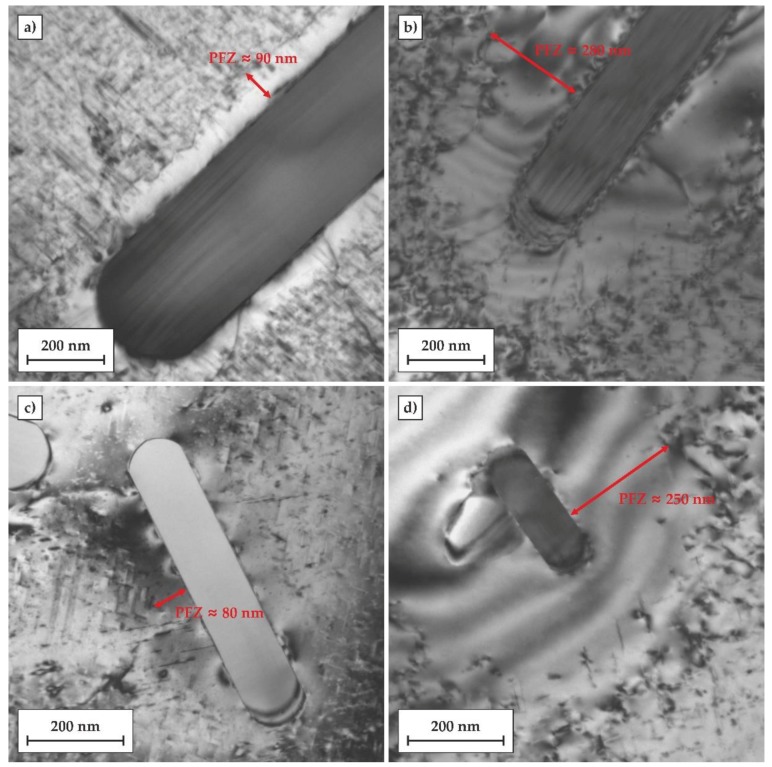
TEM micrographs showing second phase particle regions of the alloy after two investigated heat treatments: (**a**,**c**) conventional heat treatment to the T6 temper including artificial ageing at 170 °C for 24 h; (**b**,**d**) quenched directly to the temperature of 170 °C followed by 24 h of artificial ageing (T6DA).

A similar difference in the width of PFZs can be observed in the TEM micrographs of regions in close vicinity to rod-like, Fe-rich second phase particles (SPPs), as is shown in Figure 10. In the T6 temper ,the width of PFZs near the SPPs was in the range between 50 and 100 nm, while in the T6DA temper, the width of PFZs reached about 250–300 nm. In addition, other particles in the vicinity of SPPs were observed at their interface with the matrix. This may suggest that some solute atoms diffused to the SPP during ageing.

## 4. Discussion

In the conventional T6 heat treatment, the alloy is initially subjected to the solution heat treatment and then quenched in water. This allows not only for dissolving the Mg_2_Si second phase particles, but also for providing an excess of vacancies, which in turn, actively participate in the formation of the metastable phases during ageing. After water quenching, solute clusters in the supersaturated matrix are formed during natural ageing at room temperature. These clusters, in turn, are preferred nucleation sites for the main hardening phase, β”, that is formed during further artificial ageing [28,29,30,31,32]. This leads to the formation of a very fine and uniformly-distributed β” phase in the alloy matrix with narrow precipitate-free zones at the grain boundaries, as well as at large second phase particles, providing the highest level of mechanical properties. 

In the other investigated heat treatment procedure, in which the alloy is quenched directly to the artificial ageing temperature, the duration of cooling plays a crucial role. During relatively slow cooling using forced air, the supersaturated α-Al matrix is exposed to temperatures where the excessive vacancies diffuse to grain boundaries and second phase particles, leading to the formation of relatively wide precipitate-free zones after ageing. The microstructure formed during this slow cooling influences the macroscopic properties, especially electrical resistivity. Comparing electrical resistivity just after two hours of artificial ageing, it was found that the DA procedure provides significantly lower electrical resistivity. This may be associated with the formation of a much larger and widely-dispersed β” hardening phase at the same ageing temperature. The above findings can be successfully utilized in designing a heat treatment technology of the alloys for which the electrical performance is the primary factor.

## 5. Conclusions

(1) The cooling by the flowing air stream of the EN AW 6101 alloy directly from the solutionizing temperature to the temperature of artificial ageing achieves a unique combination of hardness and electrical resistivity. 

(2) The large Mg_2_Si second phase particles, produced from melt, dissolve during the 2-h solution heat treatment, and after prolonged exposure to elevated temperatures during slow cooling in the direct ageing (DA) procedure, they were not formed in the alloy microstructure.

(3) The direct ageing (DA) procedure produces a smaller number density of β″ precipitates and relatively wide precipitate-free zones along grain boundaries compared to the conventional T6 heat treatment. 

## Figures and Tables

**Table 1:** Chemical composition of EN AW 6101 alloy investigated (wt. %).

Al	Fe	Si	Cu	Zn	Ti	Mn	Mg	Ni	Sn	Pb	Cr	V	Zr	B
bal.	0.211	0.502	0.001	0.002	0.008	0.001	0.512	0.005	0.001	0.001	0.001	0.01	0.002	0.003
